# Supplementary material for: Distributed feature representations of natural stimuli across parallel retinal pathways
Source: Nat Commun. 2024 Mar 1;15:1920. doi: 10.1038/s41467-024-46348-y (PMC10907388; doi:10.1038/s41467-024-46348-y)
Supplement: Supplementary file 1 — Supplementary Information [file 41467_2024_46348_MOESM1_ESM.pdf]

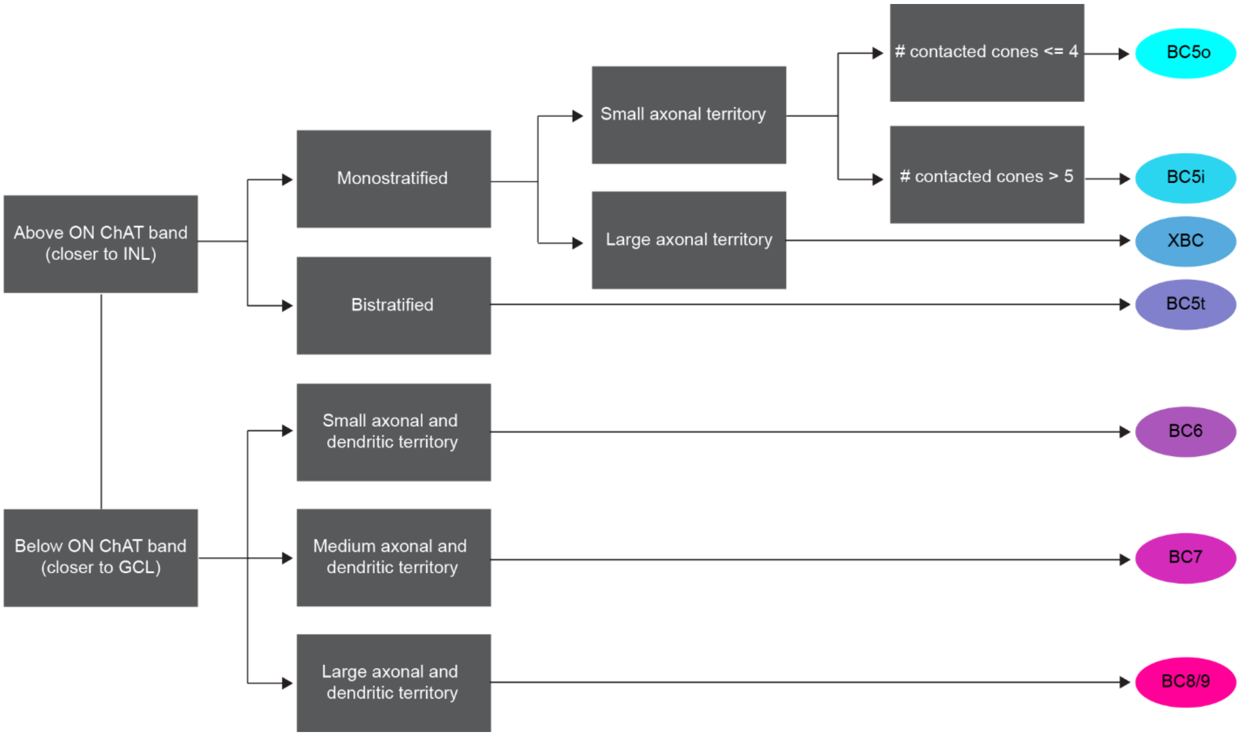

Supplementary Fig. 1 | Morphological classification of ON bipolar cells. Illustration of the morphological classification tree used to separate ON cone bipolar cells into seven distinct types. The decision criteria encompass IPL targeting, stratification patterns, dendritic and axonal territories, and the number of cone photoreceptors contacted by the bipolar cell dendrites.

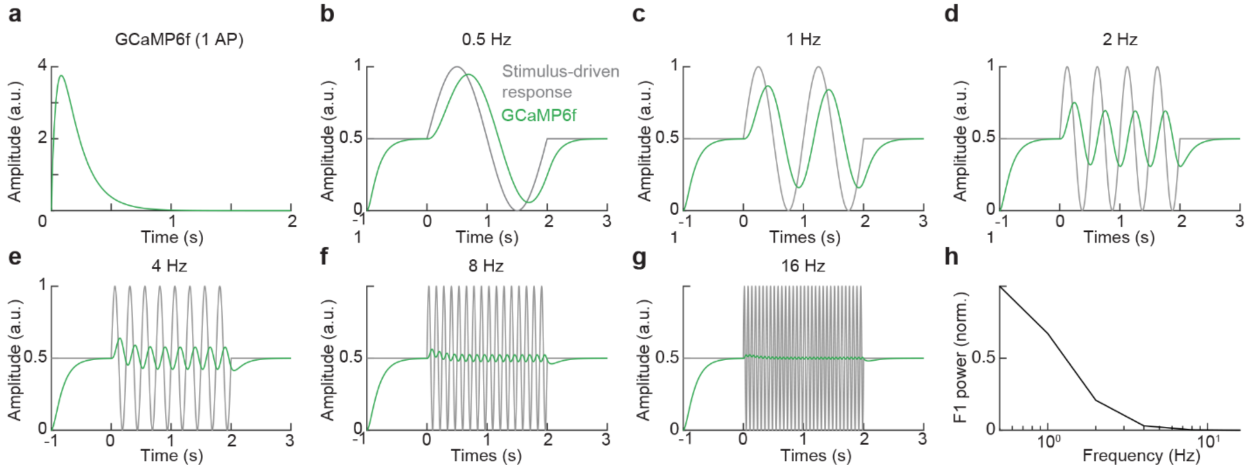

Supplementary Fig. 2 | Simulation of non-spiking GCaMP6f signals to frequency modulation. a GCaMP6f response dynamics are captured by its decay and rise time constants ($\tau_{1/2}$), set at 156 ms and 49.5 ms, respectively. These constants, initially reported by Chen et al. [1], are adjusted to reflect the recording temperature's influence, with the specifics of this temperature correction elucidated by Ohkura et al. [2]. Chen et al. [1] based their measurements on the signal elicited by a single action potential (AP) in the primary visual cortex of mice. **b-g** Simulated GCaMP6f responses (green) to varied frequency modulations (ranging from 0.5 Hz to 16 Hz, gray). These simulations approximate the non-spiking bipolar cells' membrane potentials. The resulting signals were derived by convolving the frequency modulation with the GCaMP6f response curve presented in (**a**). **h** F1 power normalized to its peak (attributed to the 0.5 Hz frequency) plotted against frequencies on a $\log_{10}$ scale. Source data for this figure are provided as a Source Data file.

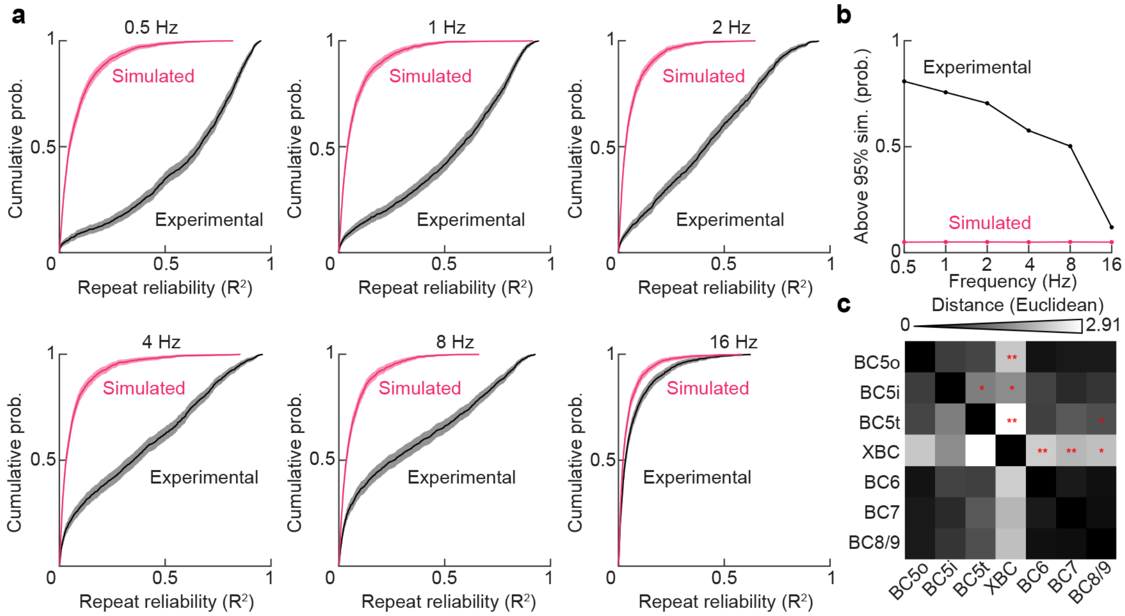

Supplementary Fig. 3 | Quality control of flicker response detection. a Cumulative probability distributions of the repeat reliabilities of all ROIs (n=1,413) segmented from 42 recorded BCs. Control data (simulated, red) were generated by estimating autoregressive parameters and producing response traces not entrained to visual stimuli but maintaining the same temporal correlation as the experimental data (black). The cumulative distributions were calculated and simulated separately for each frequency from 0.5 to 16 Hz. **b** We set a threshold based on the 9th percentile of the simulated data and measured the proportion of ROIs exceeding that threshold. At all stimulus frequencies except 16 Hz, more than 50% of ROIs significantly exceeded the threshold set from the simulated data (82% at 0.5 Hz, 75% at 1 Hz, 70% at 2 Hz, 55% at 4 Hz, and 52% at 8 Hz). **c** Paired Euclidean distances between cell type F1 powers at 2, 4, and 8 Hz are color-coded at the top. By bootstrapping, the statistical significance in the upper triangle is denoted by asterisks (*$p < 0.05$, **$p < 0.01$, ***$p < 0.001$, with FDR correction). Note that only XBC and BC5t significantly differ from the other types. Source data for this figure are provided as a Source Data file.

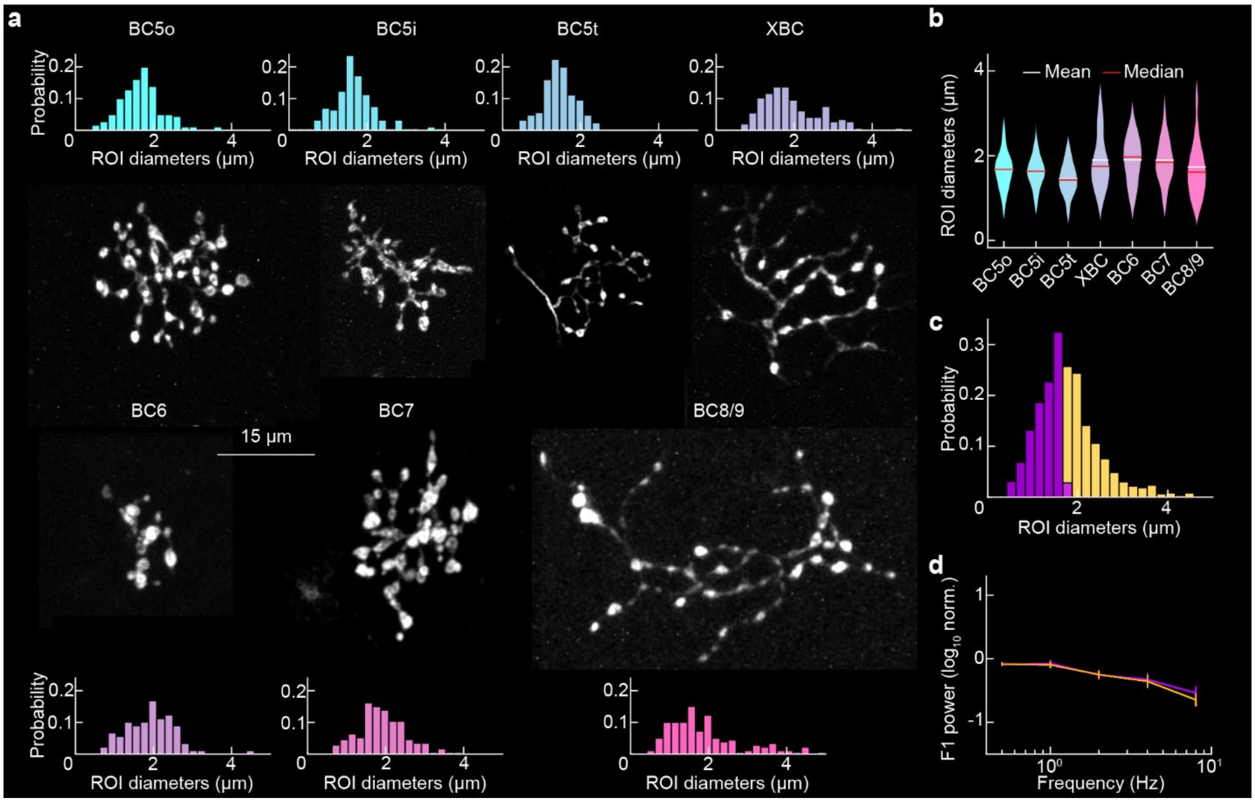

Supplementary Fig. 4 | ROI size and temporal filtering in ON bipolar cell axons. a Representative axon images of the different ON bipolar cell types (scale bar: 15 μm) are shown in the center of the panel. Surrounding this, the distributions of ROI diameters (threshold: minimum ROI size of 5 pixels and repeat reliability > 0.1) measured from two-photon image stacks of all cell types are plotted. The numbers of ROIs for each type is 180 (BC5o), 186 (BC5i), 125 (BC5t), 146 (XBC), 86 (BC6), 173 (BC7), and 179 (BC8/9). ROI diameters of XBC (i.e., the most transiently responding type) did not differ significantly from other types (Kruskal-Wallis test with multiple comparison correction). ROI diameters of BC5t were significantly smaller than all other types ($p < 0.05$ for BC5i, $p < 0.01$ for BC5o, and $p < 0.001$ for the rest). BC6 and BC7 ROIs were significantly larger than BC5o ($p < 0.05$), BC5i ($p < 0.05$ for BC6 and $p < 0.01$ for BC7), and BC5t. **b** A violin plot illustrates the ROI diameter distributions (means indicated by white lines, medians by red lines). **c** Shows the distribution of ROI diameters from combined data. In (**d**), a median ROI diameter of 1.68 μm divides ROIs into two groups for testing: purple indicates the smaller size group and yellow the larger ($n = 539$ for each). **d** Summary plot comparing the temporal frequency-response functions of small (purple) and large (yellow) ROIs. Tested with two-way ANOVA, no statistical significance was found for size ($p = 0.56$) or the interaction between frequency and size ($p = 0.94$), although the main effect of frequency was significant ($p < 0.001$). The number of cells in the small size group is 7 (BC5o), 7 (BC5i), 4 (BC5t), 6 (XBC), 6 (BC6), 5 (BC7), and 5 (BC8/9). The number of cells in the large size group is 7 (BC5o), 7 (BC5i), 4 (BC5t), 7 (XBC), 5 (BC6), 6 (BC7), and 5 (BC8/9). Source data for this figure are provided as a Source Data file.

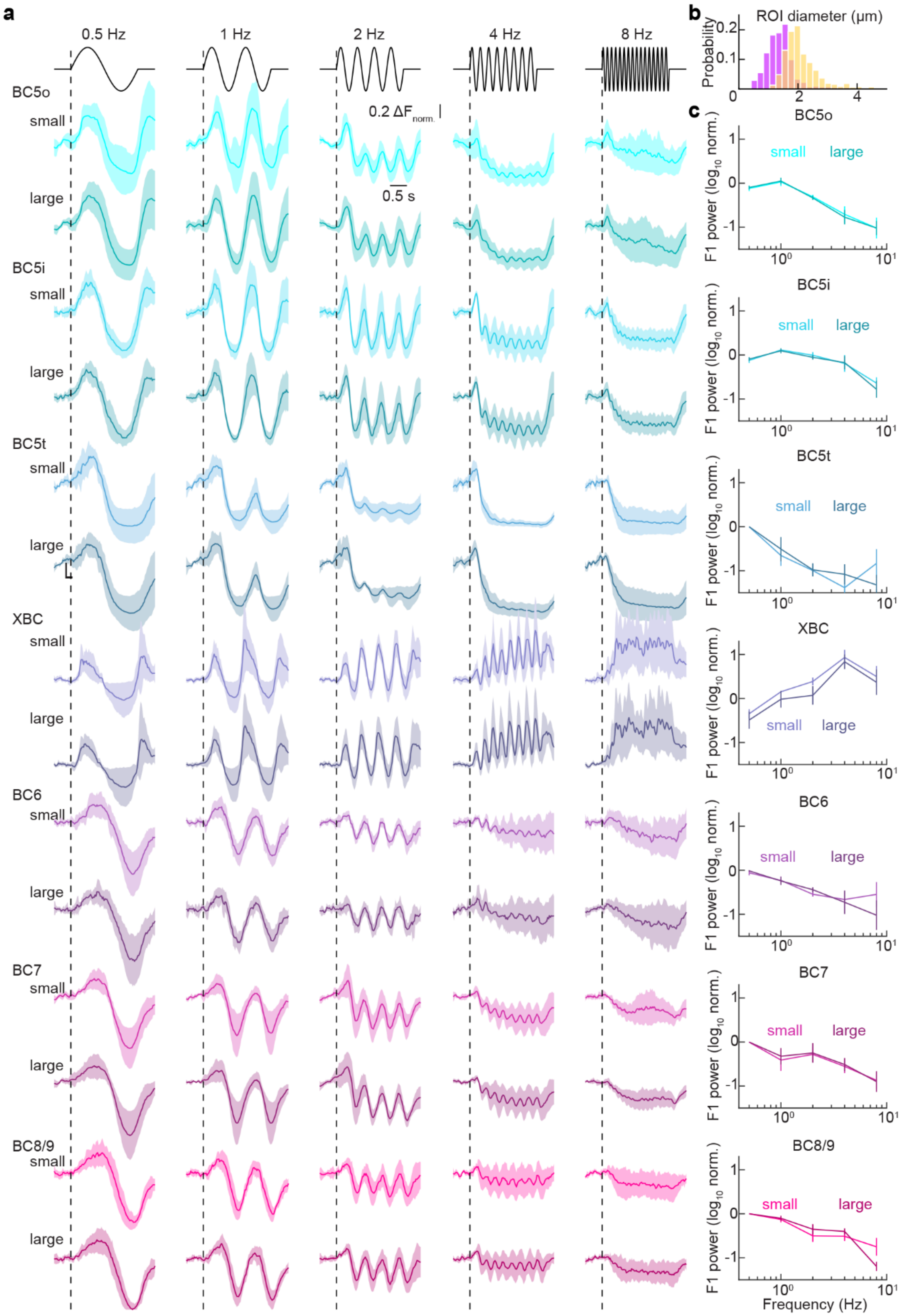

Supplementary Fig. 5 | ROI size and temporal filtering within ON bipolar cell types. a Averaged traces of ON bipolar cell type responses to different frequency stimuli in a 150 µm

diameter spot (see Fig. 2). The SEM is indicated by the shaded area around the trace. For each recorded cell, we divided ROIs into two groups based on their diameters. Different cell types are color-coded. For each cell type, the second row represents the ROI group with larger diameters, indicated by darker shades. Cell type counts are as follows: BC5o (n = 7), BC5i (n = 7), BC5t (n = 4), XBC (n = 7), BC6 (n = 6), BC7 (n = 6), and BC8/9 (n = 5). **b** Probabilistic distribution of all ROI diameters based on the split per recording. The larger ROI diameter group is indicated in yellow, and the smaller group in purple. **c** Summary plots of F1 power for each bipolar cell type, where data from individual cells are divided into smaller and larger ROI-size groups, indicated by lighter and darker colors, respectively. We observed no statistically significant difference between ROI-size groups of any cell type, nor in the interaction with frequencies. The main effects of frequencies are significant for all ON bipolar cell types ($p < 0.001$). Source data for this figure are provided as a Source Data file.

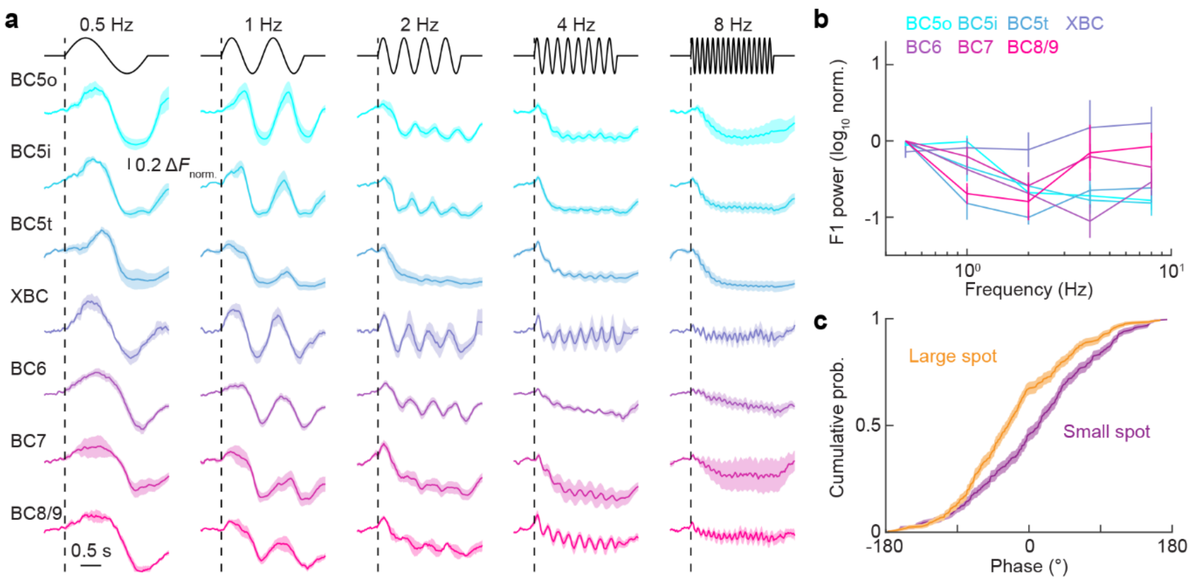

Supplementary Fig. 6 | Frequency responses of ON bipolar cells to large spots. a ON bipolar cell types were subjected to an 800 µm diameter spot undergoing sinusoidal contrast fluctuations across various frequencies, each lasting for 2 s. The cell types, differentiated by unique colors, and their respective sample sizes are: BC5o (n = 7), BC5i (n = 7), BC5t (n = 4), XBC (n = 7), BC6 (n = 6), BC7 (n = 6), and BC8/9 (n = 5). Shaded areas indicate the mean ± SEM for each type. **b** Summary data of the F1 power across a frequency range (0.5 – 8 Hz) for the 800 µm spot. The F1 powers were normalized to the maximum frequency responses and corrected for contributions of the GCaMP6f indicator kinetics (see Supplementary Fig. 2 and Methods for details). Line plots represent the mean, and error bars delineate the ±SEM range. The sample sizes mirror those in (**a**). **c** Cumulative distributions of phase values, derived from the Fourier transformation of the F1 component for each modulation frequency. The phase shifts in response to two distinct spot sizes, 150 µm (purple) and 800 µm (orange), are compared. The shaded regions indicate the mean ± SEM for each spot size. Notably, a significant phase difference between the two spot size responses is observed (two-sample Kolmogorov-Smirnov test, $p < 0.001$, $d = 0.27$), with identical sample sizes for both conditions ($n = 210$). Source data for this figure are provided as a Source Data file.

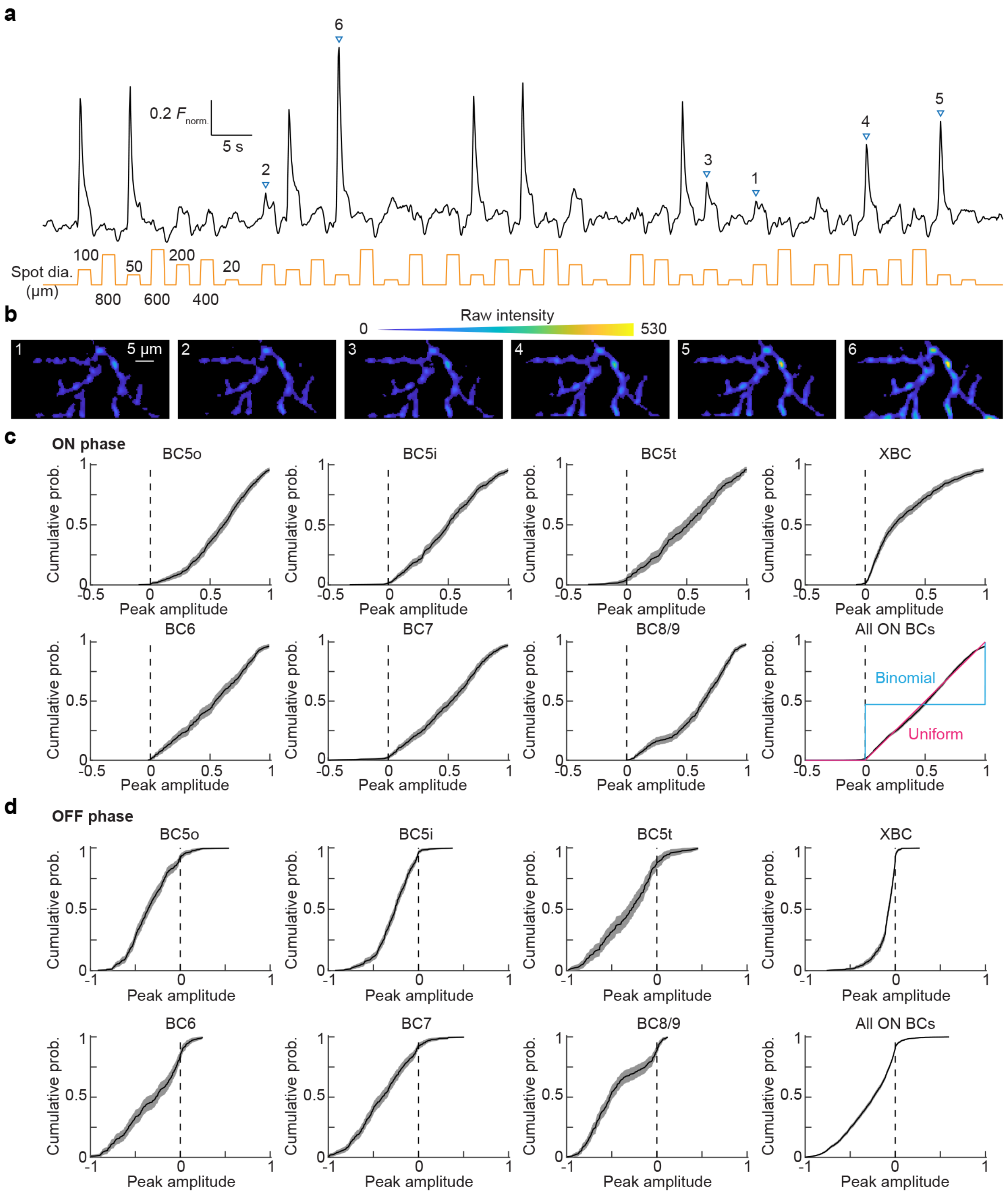

Supplementary Fig. 7 | No evidence for spikes in ON bipolar cell axons. a Representative XBC responses to spot stimuli with diameters ranging from 20 to 800 µm. The responses are shown in black at the top and stimulus sizes are indicated by the orange trace below. The trace consists of five blocks, each block representing a different pseudorandom sequence of seven spot sizes. Blue triangles highlight six distinct response events. The two-photon images corresponding to these peaks are shown in (**b**). **b** Two-photon images of six peaks from (**a**) illustrate the averaged raw recording intensity at three time points: immediately before, at the

peak intensity, and right after the peak. The intensities are color-coded as indicated above the images. **c** Cumulative distributions of peak amplitudes for each ON trial (0.1 - 1.5 s post-stimulus onset), for trials with a repeatability exceeding 0.5. The 95% confidence intervals are shown as shaded areas. Results for all ON bipolar cell types are presented in the first seven panels, with the eighth panel aggregating results across all cell types. Cumulative probabilities of binomial and uniform distributions are represented in sky blue and magenta, respectively. This analysis included 3,215 trials from 49 BCs, identifying 3090 peak events. The subpanel data quantify the peak amplitudes, with the number of trials for each type as follows: 435 (BC5o), 510 (BC5i), 246 (BC5t), 568 (XBC), 394 (BC6), 489 (BC7), 448 (BC8/9), and 3,090 (all). **d** Cumulative distributions of peak amplitudes during the stimulus OFF phase, based on 3,215 trials with 2,418 peaks detected. The results quantify the peak amplitudes. Numbers of trials are 344 (BC5o), 447 (BC5i), 174 (BC5t), 546 (XBC), 249 (BC6), 377 (BC7), 281 (BC8/9), and 2,418 (all). Panels are organized by individual cell types and combined types.

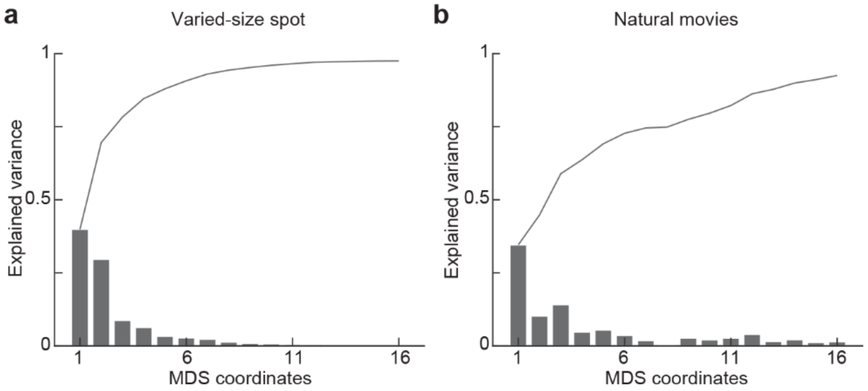

Supplementary Fig. 8 | Variance explained by multidimensional scaling (MDS) coordinates. **a** Bar chart depicting the variance contributed by different MDS coordinates to the bipolar cell response elicited by artificial stimuli. The cumulative explained variance, starting from the first coordinate, is overlaid as a gray line. This analysis incorporates 290 response units. **b** Analogous to (**a**), this panel illustrates the encoding space derived from ON bipolar cell response variance to naturalistic movies. Here, 185 response units were evaluated. Source data for this figure are provided as a Source Data file.

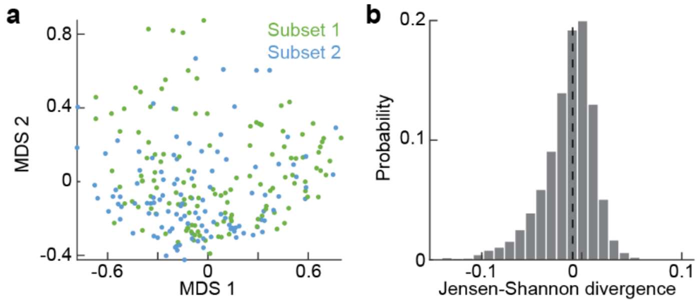

Supplementary Fig. 9 | Batch effect evaluation on the encoding space. a Scatter plot showing the distribution of randomly divided ON bipolar cell subsets within the encoding space. The x and y axes represent the first and second MDS coordinates, respectively. The Jensen-Shannon divergence (JSD) between these subsets is 0.0378. **b** The probability distribution of JSDs between subsets split based on recordings and their corresponding randomly allocated datapoint subsets. Notably, these differences were not statistically significant (Permutation test, $p = 0.32$). The analysis encompasses 15 distinct recordings from seven mice. The assessment contrasts the distribution from 2,000 random samples (out of a potential 6,435 recording split combinations) against 20,000 resamples (ten times the number) of random datapoint allocations. The latter control set is designed to match the number of datapoint splits in the recording subsets. A median difference of -0.0089 is indicated by a dashed black line. Source data for this figure are provided as a Source Data file.

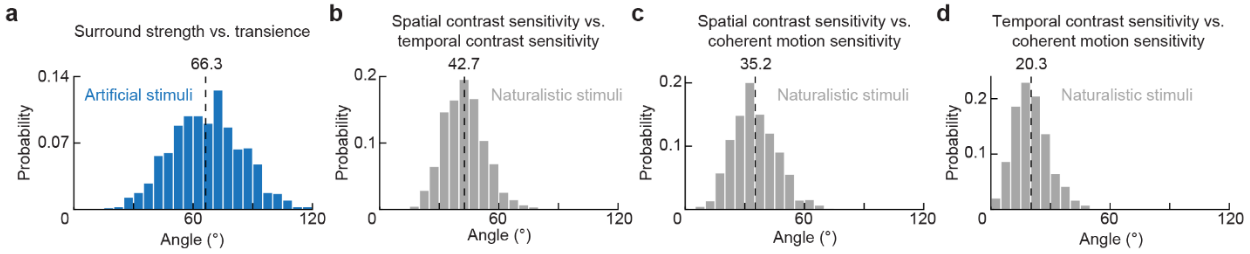

Supplementary Fig. 10 | Angular deviation among visual features in the encoding space. a Angular dispersion between the vector sums of paired sub-sampled sets for surround strength and response transience of ON bipolar cells (stimulus: spots of varying size). Each sub-sampling incorporates 90 paired vectors, 0.21% of all combinations. The presented distribution is the result of 1,000 iterations of sub-sampling. **b** Analogous to (**a**), focusing on the angular deviation between the spatial and temporal contrast sensitivities of ON bipolar cells (stimulus: naturalistic movies). Every sub-sample incorporates 90 paired vectors, 0.53% of all combinations. **c** Analogous to (**b**) for angular dispersion of spatial contrast sensitivity and coherent motion sensitivity. **d** Analogous to (**b**) for angular dispersion of temporal contrast sensitivity and coherent motion sensitivity.

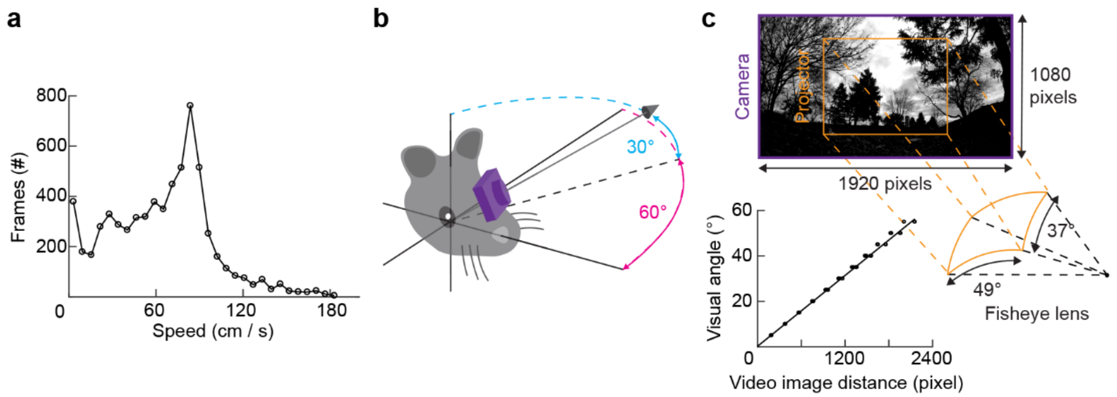

Supplementary Fig. 11 | Acquisition of naturalistic movies for mouse vision with distortion correction. a Graph of the distribution of camera movement speeds across all frames in the 11 movie clips, calculated 6.2-cm/s bins. These speed ranges align with the four recognized gaits of mouse locomotion: walk, trot, gallop, and bound [3]. **b** Schematic representation of a fixed gaze in relation to head positioning. The camera (indicated in purple) was attached via an adjustable gooseneck clamp to a movable support frame. This setup permitted the camera to achieve angles close to 60° (magenta) for azimuth and 30° (blue) for elevation, mirroring the natural fixation points of mice [4]. **c** Calibration procedure for retinal projection from the fisheye lens of the camera. Top shows a single movie frame (outlined in purple). By assessing the relationship between the physical angle from the camera and the associated pixel distance, a linear association is maintained (as illustrated in the bottom-left scatter plot). Considering that the projection plane on the mouse's retina is 1,188 x 1,584 μm^2, we utilized the relationship between visual angles and image pixel distance to extract a region (highlighted in orange) that represents a 37° x 49° segment of the mouse's visual field.

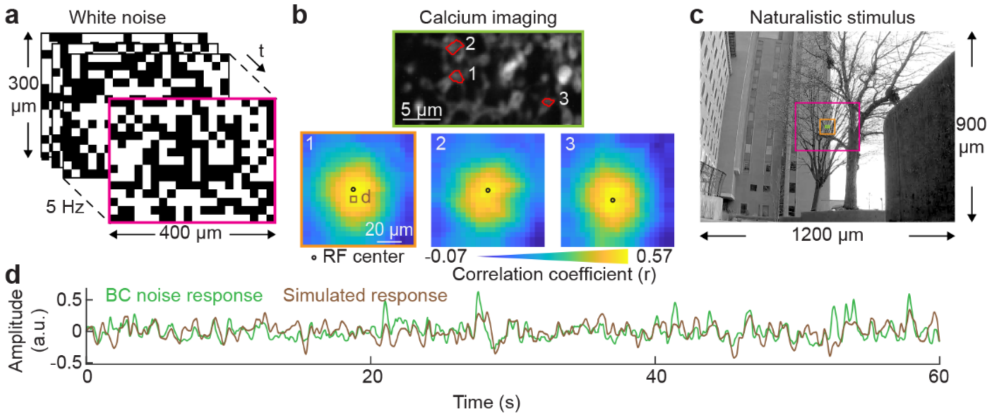

Supplementary Fig. 12 | Receptive field mapping for in-center contrast analysis. a Illustration of the binary spatially jittered binary checkerboard (each check: 20 x 20 μm^2) white noise stimulus (frame rate: 5 Hz, duration: 3 min). **b** Top: Diagram of the process used to determine the receptive field center of each ROI. Three morphologically segmented ROIs (demarcated in red) are shown in the standard deviation image of the GCaMP6f timeseries (outlined in green). Bottom: The maps illustrate correlation coefficients between ROI responses and simulated responses for receptive fields with a diameter of 80 μm centered on 440 different locations. Locations were uniformly spaced, 4 μm apart in both the x and y axes. The first map is highlighted in orange. An example of the correlation between observed and simulated responses is shown in (**d**). The corresponding ROI of the top illustration is labeled in the top left corner. The receptive field center locations were defined as the position where the correlation coefficient between observed and simulated responses, inferred via a spline 2D interpolation from the grid correlation coefficient as described. **c** Illustration of the differences in scales: the imaging area is highlighted in green (as shown in (**b**)), the map of the correlation coefficient in orange (as in (**b**)), with the projected natural movie frame in the background. Dimensions of the image are included at the bottom and on the right for clarity.

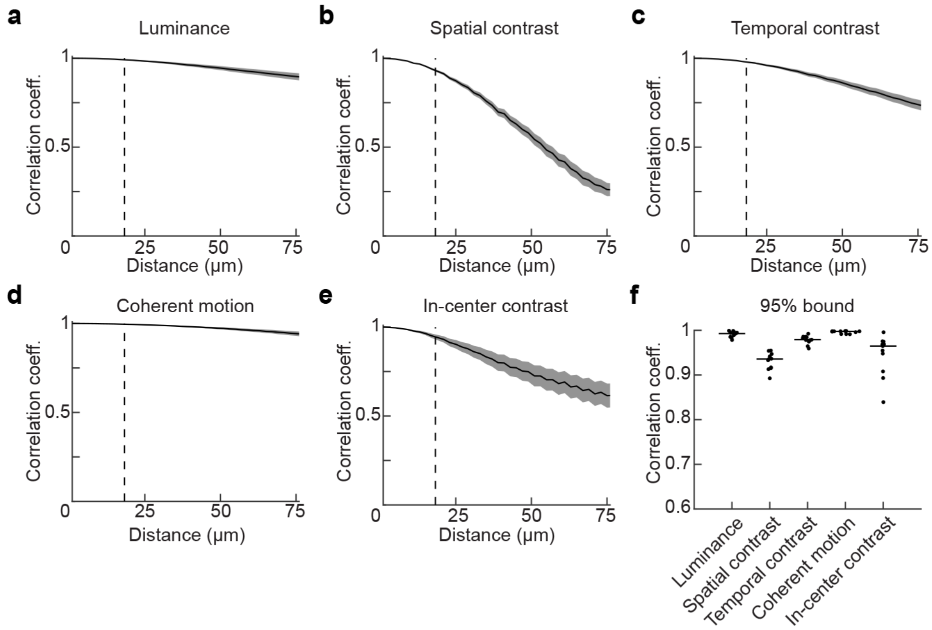

Supplementary Fig. 13 | Spatial correlation of visual features in naturalistic movies. a The correlation of luminance across various distances among spatially separated receptive fields (RFs). Data is derived from 11 naturalistic movie clips, with the shaded region indicating the standard error of the mean. Correlation values are the median within 2 µm bins. The dashed line marks the distance (18.3 µm) within which 95% of receptive fields are contained, as determined from white noise stimulus recordings (n = 59) with an explained variance exceeding 0.1; the standard deviation of this RF distribution is 9.94 µm. **b-e** Analogous to (**a**), but spatial contrast (**b**). temporal contrast (**c**), coherent motion (**d**), and in-center contrast (**e**). **f** Summary plot of correlation coefficients at the 95% boundary for all visual features, with individual dots representing different movie clips. Source data for this figure are provided as a Source Data file.

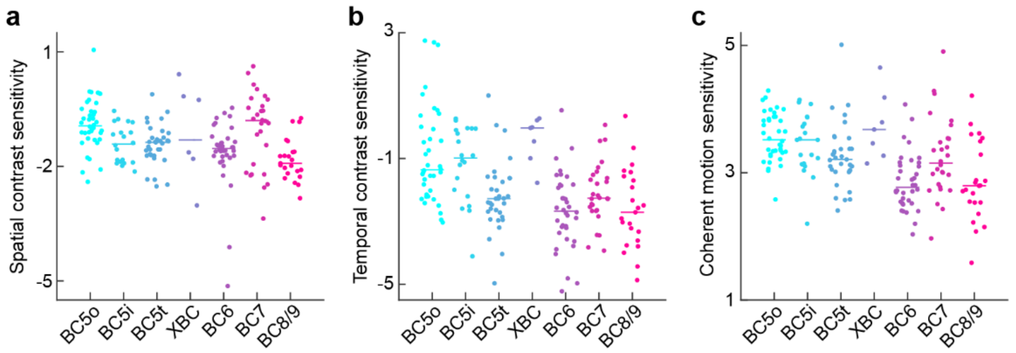

Supplementary Fig. 14 | Cell-type-specific differences in feature sensitivities among ON bipolar cells. a Swarm plot showing the spatial contrast sensitivity of different ON bipolar cell types. Each type is shown in a unique color, labeled on the x-axis. Sample sizes as follows: BC5o (n = 38), BC5i (n = 20), BC5t (n = 33), XBC (n = 7), BC6 (n = 36), BC7 (n = 28), BC8/9 (n = 23). **b** Analogous to (**a**) for temporal contrast sensitivities. **c** Analogous to (**a**) for coherent motion sensitivities. Source data for this figure are provided as a Source Data file.

Supplementary references

1. Chen, T.-W. *et al.* Ultrasensitive fluorescent proteins for imaging neuronal activity. *Nature* **499**, 295–300 (2013).
2. Ohkura, M. *et al.* Genetically encoded green fluorescent Ca2+ indicators with improved detectability for neuronal Ca2+ signals. *PLoS One* **7**, e51286 (2012).
3. Bellardita, C. & Kiehn, O. Phenotypic characterization of speed-associated gait changes in mice reveals modular organization of locomotor networks. *Curr. Biol.* **25**, 1426–1436 (2015).
4. Denman, D. J. *et al.* Mouse color and wavelength-specific luminance contrast sensitivity are non-uniform across visual space. *Elife* **7**, (2018).
