# Supplementary material for: Distributed feature representations of natural stimuli across parallel retinal pathways
Source: Nat Commun. 2024 Mar 1;15:1920. doi: 10.1038/s41467-024-46348-y (PMC10907388; doi:10.1038/s41467-024-46348-y)
Supplement: Supplementary file 3 — Reporting Summary [file 41467_2024_46348_MOESM3_ESM.pdf]

nature portfolio

Corresponding author(s): Daniel Kerschensteiner

Last updated by author(s): Jan 26, 2024

Reporting Summary

Nature Portfolio wishes to improve the reproducibility of the work that we publish. This form provides structure for consistency and transparency in reporting. For further information on Nature Portfolio policies, see our Editorial Policies and the Editorial Policy Checklist.

Statistics

For all statistical analyses, confirm that the following items are present in the figure legend, table legend, main text, or Methods section.

n/a	Confirmed	
☐	☒	The exact sample size (*n*) for each experimental group/condition, given as a discrete number and unit of measurement
☐	☒	A statement on whether measurements were taken from distinct samples or whether the same sample was measured repeatedly
☐	☒	The statistical test(s) used AND whether they are one- or two-sided<br>*Only common tests should be described solely by name; describe more complex techniques in the Methods section.*
☒	☐	A description of all covariates tested
☐	☒	A description of any assumptions or corrections, such as tests of normality and adjustment for multiple comparisons
☐	☒	A full description of the statistical parameters including central tendency (e.g. means) or other basic estimates (e.g. regression coefficient) AND variation (e.g. standard deviation) or associated estimates of uncertainty (e.g. confidence intervals)
☐	☒	For null hypothesis testing, the test statistic (e.g. *F*, *t*, *r*) with confidence intervals, effect sizes, degrees of freedom and *P* value noted<br>*Give P values as exact values whenever suitable.*
☒	☐	For Bayesian analysis, information on the choice of priors and Markov chain Monte Carlo settings
☒	☐	For hierarchical and complex designs, identification of the appropriate level for tests and full reporting of outcomes
☐	☒	Estimates of effect sizes (e.g. Cohen's *d*, Pearson's *r*), indicating how they were calculated

Our web collection on statistics for biologists contains articles on many of the points above.

Software and code

Policy information about availability of computer code

Data collection	For two-photon imaging, we used a custom-built upright two-photon microscope (Scientifica) equipped with a 60X 1.0 NA water immersion objective (Olympus), and the Scanimage r3.8 toolbox in MATLAB. Data acquisition was performed using a DAQ NI PCI6110 board (National Instruments). For confocal imaging, we utilized an FV1000 laser scanning microscope (Olympus) with a 60X 1.35 NA oil immersion objective (Olympus). When recording natural movies, a commercial camera (Crosstour CT9000) was employed.
Data analysis	We used ImageJ (NIH) to process both confocal and two-photon data, particularly for characterizing each recording bipolar cell and reconstructing 3D bipolar cell skeletons. ImageJ version 1.53v was used for most analyses. MATLAB R2021a was employed for plotting the figures and performing statistical tests. MATLAB R2016b and Cogent Graphics v1.33 were used for presenting visual stimuli. Customized MATLAB code can be found in the following GitHub repository: https://github.com/Jen-Chun-Hsiang/ONBCEncoding. Adobe Illustrator (2022, v26.0.3) was used to assemble the final figures.

For manuscripts utilizing custom algorithms or software that are central to the research but not yet described in published literature, software must be made available to editors and reviewers. We strongly encourage code deposition in a community repository (e.g. GitHub). See the Nature Portfolio guidelines for submitting code & software for further information.

Data

Policy information about availability of data

All manuscripts must include a data availability statement. This statement should provide the following information, where applicable:

- Accession codes, unique identifiers, or web links for publicly available datasets
- A description of any restrictions on data availability
- For clinical datasets or third party data, please ensure that the statement adheres to our policy

Source data, including all preprocessed information for analyses, traces, and graphs in figures, is provided in a spreadsheet. For inferring IPL depth, we used published supplementary data available at doi: 10.1038/nature12346. Raw data will be shared upon request.

Research involving human participants, their data, or biological material

Policy information about studies with human participants or human data. See also policy information about sex, gender (identity/presentation), and sexual orientation and race, ethnicity and racism.

Reporting on sex and gender	not applicable
Reporting on race, ethnicity, or other socially relevant groupings	not applicable
Population characteristics	not applicable
Recruitment	not applicable
Ethics oversight	not applicable

Note that full information on the approval of the study protocol must also be provided in the manuscript.

Field-specific reporting

Please select the one below that is the best fit for your research. If you are not sure, read the appropriate sections before making your selection.

☒ Life sciences ☐ Behavioural & social sciences ☐ Ecological, evolutionary & environmental sciences

For a reference copy of the document with all sections, see nature.com/documents/nr-reporting-summary-flat.pdf

Life sciences study design

All studies must disclose on these points even when the disclosure is negative.

Sample size	We did not perform power analyses to determine the sample size for each experiment. Instead, sample sizes were selected to reflect both the standard in the field and those used in our previous studies (e.g., Hsiang et al., 2017).
Data exclusions	Exclusion of mice is described in Figure 1. Scanned bipolar cells with insufficient morphological identification, based on the criterion in Supplementary Figure 1, are excluded. The exclusions for two-photon calcium imaging are based on response quality, as described in the Methods section. Further exclusion of the augmented data was conducted based on the nearest neighbor functional similarity, also detailed in the Methods. Additionally, the data exclusion for distance-dependent response parameters is indicated in the figure or text.
Replication	Experiments were replicated as noted in the figures or text of the study. Our main results were replicated across different visual stimuli, including frequency-modulated spots, varied-size spots, and natural movies. Data collection spanned approximately two years, with no noticeable differences in the responses measured.
Randomization	The core design of this study's experiments is to compare stimulus-driven responses between different identified ON bipolar cells. Cell type expression in bipolar cells is inherently stochastic due to virus infection and Cre recombinase-mediated DNA recombination. The stimuli were presented in a pseudorandom order, where the order of the different stimuli is random within each block. Blocks are used to ensure consistent quality measures over time.
Blinding	Due to the absence of observer effects in the study's experiments, conventional blinding was not relevant. Bipolar cell type identification was masked until post-hoc immunohistochemistry, and classification followed established literature with standardized measurements and thresholds.

Reporting for specific materials, systems and methods

We require information from authors about some types of materials, experimental systems and methods used in many studies. Here, indicate whether each material, system or method listed is relevant to your study. If you are not sure if a list item applies to your research, read the appropriate section before selecting a response.

Materials & experimental systems

n/a	Involved in the study
☐	☒ Antibodies
☐	☒ Eukaryotic cell lines
☒	☐ Palaeontology and archaeology
☐	☒ Animals and other organisms
☒	☐ Clinical data
☒	☐ Dual use research of concern
☒	☐ Plants

Methods

n/a	Involved in the study
☒	☐ ChIP-seq
☒	☐ Flow cytometry
☒	☐ MRI-based neuroimaging

Antibodies

Antibodies used

Chicken anti-GFP, ThermoFisher, Cat# A10262, RRID:AB_2534023, used at 1:1000
Rabbit anti-cone arrestin (CAR), Millipore, Cat# AB15282, RRID:AB_1163387, used at 1:1000
Goat anti-choline acetyltransferase (ChAT), Millipore, Cat# AB144P, RRID:AB_11213095, used at 1:200
Donkey anti-Chicken IgY (H+L) Highly Cross Adsorbed Secondary Antibody, Alexa Fluor™ 488, ThermoFisher, Cat# A78948, RRID:AB_2921070, used at 1:1000
Donkey anti-Rabbit IgG (H+L) Highly Cross-Adsorbed Secondary Antibody, Alexa Fluor™ 568, ThermoFisher, Cat# A10042, RRID:AB_2534017, used at 1:1000
Donkey anti-Goat IgG (H+L) Cross-Adsorbed Secondary Antibody, Alexa Fluor™ 633, ThermoFisher, Cat# A-21082, RRID:AB_2535739, used at 1:1000

Validation

Antibody information and validation, and references by the manufacturers:
Chicken anti-GFP, ThermoFisher, Cat# A10262: https://www.thermofisher.com/antibody/product/GFP-Antibody-Polyclonal/A10262
Rabbit anti-cone arrestin (CAR), Millipore, Cat# AB15282: https://www.emdmillipore.com/US/en/product/Anti-Cone-Arrestin-Antibody,MM_NF-AB15282
Goat anti-choline acetyltransferase (ChAT), Millipore, Cat# AB144P: https://www.sigmaaldrich.com/US/en/product/mm/ab144p
All these antibodies were also used and validated in our previous study (Shen et al, 2020)

Eukaryotic cell lines

Policy information about cell lines and Sex and Gender in Research

Cell line source(s)

State the source of each cell line used and the sex of all primary cell lines and cells derived from human participants or vertebrate models.

Authentication

Describe the authentication procedures for each cell line used OR declare that none of the cell lines used were authenticated.

Mycoplasma contamination

Confirm that all cell lines tested negative for mycoplasma contamination OR describe the results of the testing for mycoplasma contamination OR declare that the cell lines were not tested for mycoplasma contamination.

Commonly misidentified lines
(See ICLAC register)

Name any commonly misidentified cell lines used in the study and provide a rationale for their use.

Animals and other research organisms

Policy information about studies involving animals; ARRIVE guidelines recommended for reporting animal research, and Sex and Gender in Research

Laboratory animals

All the laboratory animals used in this study, specifically house mice, were maintained in a colony within the Division of Comparative Medicine at Washington University School of Medicine. The congenic mouse strain for the Ai148 line is C57BL/6J, and the Grm6-Cre line also originated from the C57BL/6J strain. The animals were housed on a 12-hour light/dark cycle with overnight dark adaptation before the experiment and had ad libitum access to food and water. Experiments were conducted at 20-21 °C with humidity levels kept between 30% and 50%. Subretinal injections were performed at P4 to P6. The ages for recordings were 2.9 +/- 0.6 months (mean, standard deviation), as described in the Methods section and Figure 1.

Wild animals

This study did not involve wild animals.

Reporting on sex

Experiments utilized adult mice of both sexes, with an almost equal distribution, as described in the Methods section. No consistent differences were noted in the results related to the sex of the animals.

Field-collected samples

This study did not involve samples collected from the field.

Ethics oversight	All animal procedures were approved by the Animal Studies Committee of Washington University School of Medicine and performed in compliance with the National Institutes of Health Guide for the Care and Use of Laboratory Animals.

Note that full information on the approval of the study protocol must also be provided in the manuscript.
